# Brain activation associated with active and passive lower limb stepping

**DOI:** 10.3389/fnhum.2014.00828

**Published:** 2014-10-28

**Authors:** Lukas Jaeger, Laura Marchal-Crespo, Peter Wolf, Robert Riener, Lars Michels, Spyros Kollias

**Affiliations:** ^1^Sensory-Motor Systems Lab, Department of Health Sciences and Technology, Eidgenössische Technische Hochschule ZürichZürich, Switzerland; ^2^Medical Faculty, University of ZurichZurich, Switzerland; ^3^Clinic of Neuroradiology, University Hospital of ZurichZurich, Switzerland; ^4^Center of MR-Research, University Children's HospitalZurich, Switzerland

**Keywords:** stepping, lower limb, motor control, locomotion, supraspinal, fMRI, robot, MARCOS

## Abstract

Reports about standardized and repeatable experimental procedures investigating supraspinal activation in patients with gait disorders are scarce in current neuro-imaging literature. Well-designed and executed tasks are important to gain insight into the effects of gait-rehabilitation on sensorimotor centers of the brain. The present study aims to demonstrate the feasibility of a novel imaging paradigm, combining the magnetic resonance (MR)-compatible stepping robot (MARCOS) with sparse sampling functional magnetic resonance imaging (fMRI) to measure task-related BOLD signal changes and to delineate the supraspinal contribution specific to active and passive stepping. Twenty-four healthy participants underwent fMRI during active and passive, periodic, bilateral, multi-joint, lower limb flexion and extension akin to human gait. Active and passive stepping engaged several cortical and subcortical areas of the sensorimotor network, with higher relative activation of those areas during active movement. Our results indicate that the combination of MARCOS and sparse sampling fMRI is feasible for the detection of lower limb motor related supraspinal activation. Activation of the anterior cingulate and medial frontal areas suggests motor response inhibition during passive movement in healthy participants. Our results are of relevance for understanding the neural mechanisms underlying gait in the healthy.

## Introduction

Sequentially coordinated periodic extension and flexion movements of the hips, knees, and ankles are common to a number of human locomotor movements, such as ground level walking, running or stair climbing. It is believed that the required sensorimotor control enabling these periodic movements is achieved by the interaction of proprioceptive feedback, the central pattern generators at the spinal level, and higher-level control signals from cortical and subcortical supraspinal centers (Duysens and Van De Crommert, 1998; Dietz, 2003; La Fougere et al., 2010). Recent findings from neuro-imaging studies indicate that the supraspinal areas might be involved in the control of gait to a higher extent than previously assumed (Miyai et al., 2001; Gwin et al., 2011).

To date, several studies have recorded neural activity from the brain while healthy participants walked on a treadmill at steady speed. A functional near infrared spectroscopy (fNIRS) study reported significant signal increases in medial primary sensorimotor regions (M1/S1) and the supplementary motor area (SMA) (Miyai et al., 2001). Similarly, electroencephalography (EEG) studies in humans (Presacco et al., 2011, 2012) and intracranial recordings in primates (Fitzsimmons et al., 2009) indicated a high involvement of a fronto-posterior cortical network in the control of walking. Further, it was demonstrated that bilateral electro-cortical activity in M1/S1, anterior cingulate cortex (ACC) as well as in the parietal cortex is dependent on the gait cycle phase (Gwin et al., 2011). Involvement of subcortical structures (i.e., the cerebellar vermis) has also been reported in response to steady-state ground level walking measured by single photon emission tomography (Fukuyama et al., 1997) or positron emission tomography (La Fougere et al., 2010) acquired subsequent to task execution.

The superior spatial resolution and the ability to image the entire brain are potential advantages of functional magnetic resonance imaging (fMRI) for investigating the supraspinal involvement in the control of upright gait. The results reported by the experiments in the erect posture using radiotracers, fNIRS or EEG are in strong agreement with the supraspinal activations found in fMRI experiments of the lower limbs, despite the absence of vestibular stimulation and body-balance in fMRI experiments due supine position of subjects during scanning. The accordance of findings across lower limb motor control experiments using different body postures and imaging modalities implies that supine fMRI experiments of the lower limbs provide information representative of gait-related brain activation. Using fMRI, active (i.e., movement generated by the participant) or passive (i.e., movement generated by an experimenter or an actuated external device) single-joint movements of the ankle or the knee (Dobkin et al., 2004; Sahyoun et al., 2004; Ciccarelli et al., 2005; Kapreli et al., 2006; Francis et al., 2009; Toyomura et al., 2012), pedaling (Christensen et al., 2000; Mehta et al., 2009, 2012), pseudo-gait (Martinez et al., 2014) and gait imagination (Malouin et al., 2003; Jahn et al., 2004, 2008; Iseki et al., 2008) commonly yielded involvement of M1/S1, secondary somatosensory cortex (S2), and SMA. Subcortical activations were seen in the cerebellum and the basal ganglia (i.e., the putamen), Generally, it is reported that passive movements, as compared to active ones, elicit weaker peak activations in the subcortico-cortical sensorimotor network (Christensen et al., 2000; Dobkin et al., 2004; Ciccarelli et al., 2005) and increased activation in the ACC, in the precuneus as well as in the right premotor cortex (Sahyoun et al., 2004). However, a regions-of-interest (ROI) analysis comparing the level of activation in specific areas of the brain by Mehta et al. (2012) was unable to detect significant differences between active and passive pedaling movements.

Other studies investigating active and passive lower limb motor control did not report direct comparisons between active and passive lower limb movements, presumably because it is challenging to precisely standardize and control motor behavior across movement conditions. Both active and passive movement execution are of particular relevance in lower limb neuro-rehabilitation. A better understanding of the effects of afferent sensorimotor cues induced by passive movement is essential for investigating the functional status and recovery potential of paretic patients unable to voluntarily activate their lower limb muscles (Ciccarelli et al., 2005). Their capacity for sensory adaptation to gait-training may be reflected in the neural activations associated with passive movement execution (Dobkin et al., 2004). Therefore, the work with gait-impaired patients in particular calls for a reproducible experimental procedure, allowing investigations of brain activation during standardized and controlled active and passive movements and comparisons over time. Although this necessity is often mentioned in the literature, to our knowledge there is no report about such a standardized procedure to examine multi-joint, lower limb movements.

The recently developed magnetic resonance (MR)-compatible robot MARCOS enables such highly repetitive and controlled delivery of standardized active and passive, periodic, bilateral, multi-joint lower limb stepping movements that resemble human gait (Hollnagel et al., 2011). Knee and foot movement dynamics are measured by position and force sensors and can be recorded for *post-hoc* analyses of motor performance and correlations to imaging data. Linear guides direct flexion and extension of the lower limbs along the sagittal plane of the participant. Furthermore, this robot is suited for the investigation of paretic patients, since the exoskeleton can also provide assistance-as-needed in lower limb movements (Hollnagel et al., 2013).

A well-known issue in neuro-imaging studies of lower limb motor control is task-correlated head-motion (Seto et al., 2001). Extraction of meaningful fMRI data during periodic stepping movements is hindered by task-correlated head motion associated with data acquisition during the execution of the motor task, which limits accurate anatomical localization of the signals (Friston et al., 1996; Field et al., 2000). However, the temporally sluggish behavior of the BOLD-signal allows to temporally separate task execution from image acquisition. This serial arrangement termed “sparse sampling imaging” (Hall et al., 1999; Dresel et al., 2005; Zaehle et al., 2007; Toyomura et al., 2012), is hence a promising approach to minimize the effects of task-correlated head motion. To our knowledge this has not been applied to investigate standardized active and passive lower limb motor tasks.

As a foundation for future research and clinical work with gait-impaired neurologic patients, the present study with healthy participants therefore aims (a) to demonstrate the feasibility of a novel imaging paradigm, combining the MR-compatible stepper MARCOS with a sparse temporal sampling fMRI protocol and (b) to delineate the supraspinal contribution specific to active and passive bilateral, periodic, multi-joint, lower limb motor control in healthy participants. This should provide a framework for comparison for future studies involving neurologic patients with lower limb deficits.

We hypothesize that the sparse sampling imaging protocol allows the detection of sensorimotor related cortical and sub-cortical activity in the brain, and that active control of bilateral periodic multi-joint lower limb movement elicits stronger activation of the sensorimotor network of the brain than does passive execution of the same movements.

## Materials and methods

This study was approved by the Ethics Committee of the Canton of Zurich (approval Nr. 856) and was conducted in accordance with the standards for research involving human participants defined by the Declaration of Helsinki. Before inclusion of participants it was ensured that they did not meet any of the following exclusion criteria: (1) neurological, musculoskeletal or cardiac dysfunction, (2) cardiac pacemaker, neuro-stimulator, or hearing aid, and (3) drug-abuse. All participants were informed about the aims and the course of the study and gave written consent for their participation. All data collection took place on the same scanner at the University Hospital of Zurich, Switzerland.

### Participants

Twenty-four healthy, right-handed and—footed (Elias et al., 1998) young adults were investigated during active and passive stepping. Four participants had to be excluded from further analysis due to excessive head-motion (i.e., translation of more than half voxel size in any direction). The remaining 20 participants (8 female) were on average aged 27 years (*SD* 4 years). Further demographic information about the study sample can be found in Table 1.

**Table 1:** **Anthropometric data of the study sample**.

	**Mean (*SD*)**	**Min**	**Max**
**Age (years)**	27.15 (4.28)	22	35
**Body height (m)**	1.74 (0.07)	163	189
**Body weight (kg)**	71.08 (9.63)	55	90
**WHQ**	15.15 (1.1)	12	16
**WFQ**	10.01 (4.56)	3	17

Values are groups means (*SD*), WHQ, Waterloo Handedness Questionnaire; values may range from −16 to 16; WFQ, Waterloo Footedness Questionnaire; values may range from −20 to 20; positive values represent dominance of the right side of the body in both tests.

**Figure 1:**
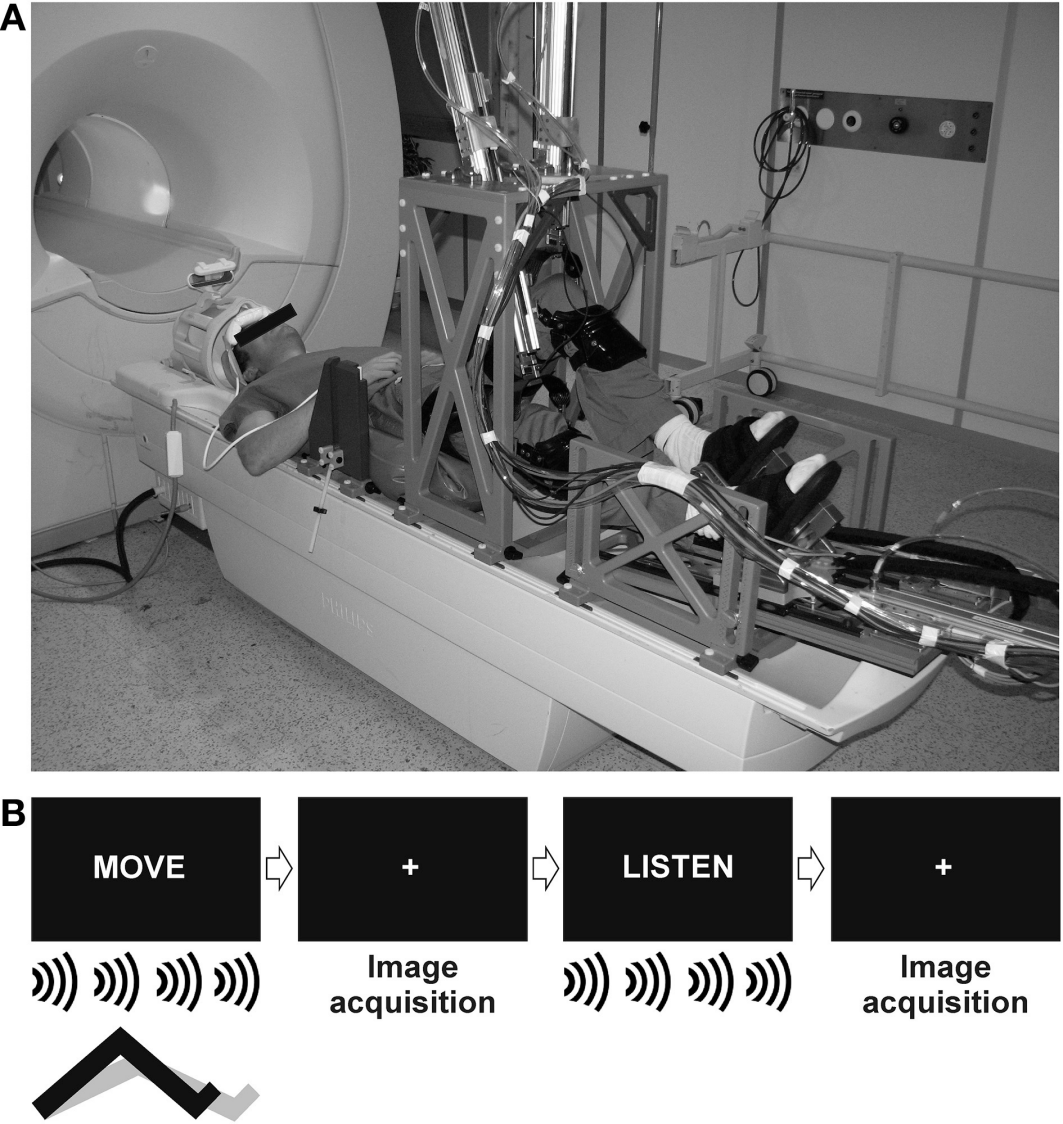
**The experimental set-up used in the study. (A)** The MR compatible stepper MARCOS was mounted to a 1.5 Tesla Philips Achieva MR-scanner (Reprinted from Hollnagel et al., 2011 with permission from Elsevier). **(B)** Movement onsets were triggered visually by the presentation of the word “MOVE.” A metronome set to 0.5 Hz was presented over the headphones to control movement frequency. Trials of movement were interleaved by an auditory control condition indicated by the word “LISTEN.” A white fixation cross was presented during image acquisition.

The pneumatic, MR-compatible, stepping robot MARCOS was used to control repetitive active and passive stepping throughout the experiment. MARCOS was designed at the Sensory-Motor Systems Lab (www.sms.hest.ethz.ch) at ETH Zurich and is built from materials of low magnetic susceptibility (i.e., aluminum, brass, polyvinyl chloride). It is a one-degree-of-freedom robotic device actuated by two pneumatic cylinders per leg allowing predefined flexion and extension movements of each leg individually in the sagittal plane. The resulting movement resembles “marching on the spot” including rotation about the hip, knee and ankle joints. Proper function of the robot is continuously monitored by several redundant mechanisms to ensure subject safety. MR-compatibility of the system was established by Hollnagel et al. (2011). Participants were secured to the robot at their knees through orthoses and their feet by the means of special shoes. Data about limb position and interaction forces between the robot and the participants were recorded by position and force sensors and stored at a frequency of 80 Hz for off-line analysis of task performance. To limit head motion, a custom made hip-fixation, a vacuum pillow at the back of the participants, shoulder belts, and an inflatable pillow (Crania, www.pearltec.ch) around the head, were combined to firmly, yet comfortably, fixate the upper body and head of each participant (Hollnagel et al., 2011). Participants could see a screen placed in front of the scanner as well as parts of the robot and their knees by the means of a mirror mounted to the head coil (Figure 1A).

### Motor paradigm

Functional imaging data during active and passive stepping were acquired in two separate runs in random order. Subjects were informed about the type of movement before the start of a run. Each run consisted of 15 trials of movement, trial duration was 10 s. In the active condition, vertical knee movement was limited to the amplitude of 0.14 m. The movement frequency was paced to 0.5 Hz by the presentation of a metronome through the earphones (Ciccarelli et al., 2005; Mehta et al., 2009), hence each leg performed five steps during each trial. In the passive condition, the same vertical knee movement amplitude (0.14 m) and frequency (0.5 Hz) were imposed to the participants by the robot in order to match movement range and velocity across conditions. The metronome was also presented during passive movements to produce corresponding auditory stimulation. Movement trials were interleaved by an auditory control condition (i.e., listen to the metronome without moving the legs) to control for auditory activations. The beginning of each trial was indicated either by the presentation of the word “MOVE” for movement trials or “LISTEN” for control trials. A white fixation cross was presented on the screen during image acquisition between the “MOVE” and “LISTEN” trials (Figure 1B) and subjects were instructed not to think about moving their legs when listening to the metronome in order to minimize effects of movement imagination or rehearsal.

Participants were familiarized with active and passive stepping inside the robot before image acquisition. During the passive movement condition, subjects should relax their legs and not engage in active leg flexion- and extension while the robot enforced a desired trajectory with predefined amplitude and frequency. In contrast, participants should voluntarily produce leg flexion and extension during the active condition while the robot followed the movement of the participant and minimized the interaction forces between the participant and the device. In this condition, the knee actuators limited the amplitude of the movement, but did not dictate the frequency. Noises of the pneumatic actuators were not audible to the participant as the valves of the actuators producing the noises were located outside the scanner room.

### Image acquisition

Imaging data were acquired on a whole body 1.5 Tesla Philips Achieva system (Philips Medical Systems, Best, The Netherlands) using an 8-channel SENSE (Pruessmann et al., 1999) head coil. The sparse sampling imaging protocol consisted of clusters of image acquisition interleaved by silent gaps of 10 s length. Each imaging cluster comprised of 3 consecutive volumes [time of repetition (*TR*) = 3.025 s]. The duration between the onsets of two imaging clusters was hence 19.075 s. Ninety three volumes in 31 clusters of 3 volumes were acquired, using a whole brain T2^*^-weighted, single-shot, echo planar imaging (EPI) sequence (time of echo = 50 ms, flip angle = 90°, SENSE factor = 1.6). Thirty five interleaved, angulated, transversal slices covering the whole brain were acquired in each volume (field of view = 220 × 220 mm, acquisition voxel size: 2.75 × 2.8 × 3.8 mm, resliced to 1.72 × 1.72 × 3.8 mm).

### Data analysis

#### Task performance

To assess task performance of the participants, the following metrics were extracted from robot position (after filtering with a 1st order Butterworth filter with a cut off frequency of 4 Hz) and force data, using custom routines written in Matlab 2012b (Mathworks, Inc., Natick, MA, USA, www.mathworks.com). For each trial the mean stepping amplitude (displacement of the knee along the linear guide), mean stepping frequency, and the mean peak robot-participant interaction force at the knee (*KF*) and foot (*FF*) were computed. Trial means were averaged across the left and the right side since stepping of the left and the right leg was not significantly different (*p*-values > 0.1). Then, trial means were averaged within each condition per participant. Each performance parameter characterizing active and passive stepping was then entered into a two-way analyses of variance (ANOVA) with the factors “trial” and “condition” to test for between-trial and between-condition effects. In addition, the root mean squared error (RMSE) for each parameter in each condition was calculated. For 95% of the measurements, the absolute deviation between a single measurement and its true value is expected to be less than the RMSE multiplied by 1.96 (Bland and Altman, 1996).

#### fMRI analysis

Functional data were analyzed using SPM8 (Wellcome Department of Cognitive Neurology, London, UK, www.fil.ion.ucl.ac.uk/spm) running on Matlab 2012b (Mathworks, Inc., Natick, MA, USA, www.mathworks.com). For each run, the three volumes prior to the first “MOVE” block were discarded. The remaining 90 images were realigned to the mean image and unwarped to account for residual head motion related variance and image distortions along air-tissue boundaries (Andersson et al., 2001). Subsequently, images were normalized to standard MNI space using the EPI template provided by the Montreal Neurological Institute (MNI brain), re-sliced to 2 × 2 × 2 mm voxel size, and smoothed using an 8 mm full-width at half-maximum Gaussian kernel. The estimated realignment parameter data were filtered using the discrete cosine transform matrix filter (cut off at 128 s) incorporated in SPM8, to remove any linear baseline drift. FMRI data sets were only included in the subsequent 1st level statistical analysis if total head displacement was below half voxel size in each dimension after filtering.

1st level statistical analysis was carried out for each participant individually by modeling the active and passive stepping condition as two separate regressors in the same general linear model (GLM) (Friston et al., 1994). The auditory control conditions were not modeled. Two additional regressors of no interest were included in the GLM for each condition to account for the T1-decay along the three consecutive volumes (Zaehle et al., 2007). A high pass filter (cut off at 128 s) was used to remove slow signal drifts. To account for the sparse-sampling fMRI scheme, data during each trial were modeled using a boxcar function [1st order, window length 3 × *TR* (i.e., 9.075 s)] (Liem et al., 2012). Contrast images were computed for: active vs. baseline and passive vs. baseline as well as active vs. passive, and passive vs. active stepping.

Each participant's contrast images from the 1st level analysis were then entered into a random effects 2nd level analysis using a One-Way ANOVA. The resulting statistical parametric maps were thresholded at a cluster-corrected voxel threshold of *p* < 0.001 (spatial extent: *k* ≥ 42 contiguous voxels) (Forman et al., 1995; Slotnick et al., 2003). The cluster size threshold for the selected *p*-values was estimated using Monte Carlo simulations (http://afni.nimh.nih.gov/pub/dist/doc/program_help/AlphaSim.html). The cluster threshold method was applied to control for the overall type I error. Anatomical correlates of clusters of activation were determined with the help of probabilistic cytoarchitectonic maps implemented in the Anatomy toolbox (Eickhoff et al., 2005).

To distinguish the differences in activation strengths between active and passive stepping in more detail, regions of interest (ROI) were built from the functional activations of a conjunction analysis (Nichols et al., 2005) between these two contrasts (voxel threshold *p* ≤ 0.001, cluster-corrected, *k* = 42 voxels). Parameter estimates (i.e., β-weights) and percent signal change were extracted from the following ROIs using the MarsBaR toolbox (Brett et al., 2002): left S2 (-50/-32/20), right S2 (46/-30/24), cerebellar vermis (0/-46/-8), right putamen (30/0/8), right lingual gyrus (14/-78/-14), right middle occipital gyrus (26/-88/16), right inferior temporal gyrus (46/-62/-4), and left inferior occipital gyrus (-52/-74/-4). An extensive cluster centered around (-2/-14/64) covering bilateral M1/S1, SMA, and CMA (5117 voxels) was manually further divided into three additional bilateral ROIs by building spheres (radius of 4 mm) using the spatial coordinates for knee movements from Kapreli et al. (2006): left M1/S1 (-14/-37/65) and right M1/S1 (16/-35/67), SMA proper left at (-2/-24/66) and right at (0/-24/68), CMA left at (-12/-6/44) and right at (10/-6/42). The extracted β-weights and percent signal change from each respective ROI were subsequently tested for significant differences across conditions using the Wilcoxon signed-ranked test (α = 0.05) as not all of the data samples were normally distributed (Shapiro-Wilk test).

To assess significant relationships between participant performance during active and passive stepping and the degree of brain activation, performance metrics were correlated to the β-weights of each ROI and Spearman's ρ were calculated (α = 0.05).

## Results

### Motor performance

A two-way ANOVA with repeated measures was conducted for each performance metric to examine the effects of trial repetition and condition. No significant effect of trial was found for any of the performance metrics (i.e., all *p*-values > 0.1). There was only a significant main effect of condition for maximal knee force [*F*_(1, 14)_ = 56.759, *p* < 0.001, η^2^_*p*_ = 0.802] and maximal foot force [*F*_(1, 14)_ = 8.519, *p* < 0.011, η^2^_*p*_ = 0.378] but not for movement amplitude and stepping frequency. RMSE values for all performance metrics were up to 5 times higher during active as compared to passive (Table 2).

**Table 2:** **Performance metrics during active or passive stepping**.

	**Passive**				**Active**				
	**Mean (*SD*)**	**Min**	**Max**	**RMSE**	**Mean (*SD*)**	**Min**	**Max**	**RMSE**	***p*-value**
Knee amplitude (m)	0.141 (0.005)	0.133	0.147	0.001	0.153 (0.022)	0.103	0.189	0.005	0.062^*^
Stepping frequency (Hz)	0.521 (0.027)	0.502	0.584	0.033	0.539 (0.030)	0.492	0.613	0.048	0.141
Maximal knee force (N)	−18.113 (12.724)	−36.799	4.028	2.006	54.408 (31.879)	11.783	121.855	10.782	0.000^**^
Maximal foot force (N)	50.110 (9.078)	39.321	71.892	0.912	65.981 (18.320)	36.226	121.266	4.475	0.011^**^

*All values are overall group means (SD), RMSE, root mean squared error; two way repeated-measures ANOVA with the factors “trial” and “condition”, p-values indicate significances for tests of main effect of condition*,

** *p* ≤ 0.05,

* *p* ≤ 0.1, α = 0.05.

### Functional data

**Figure 2:**
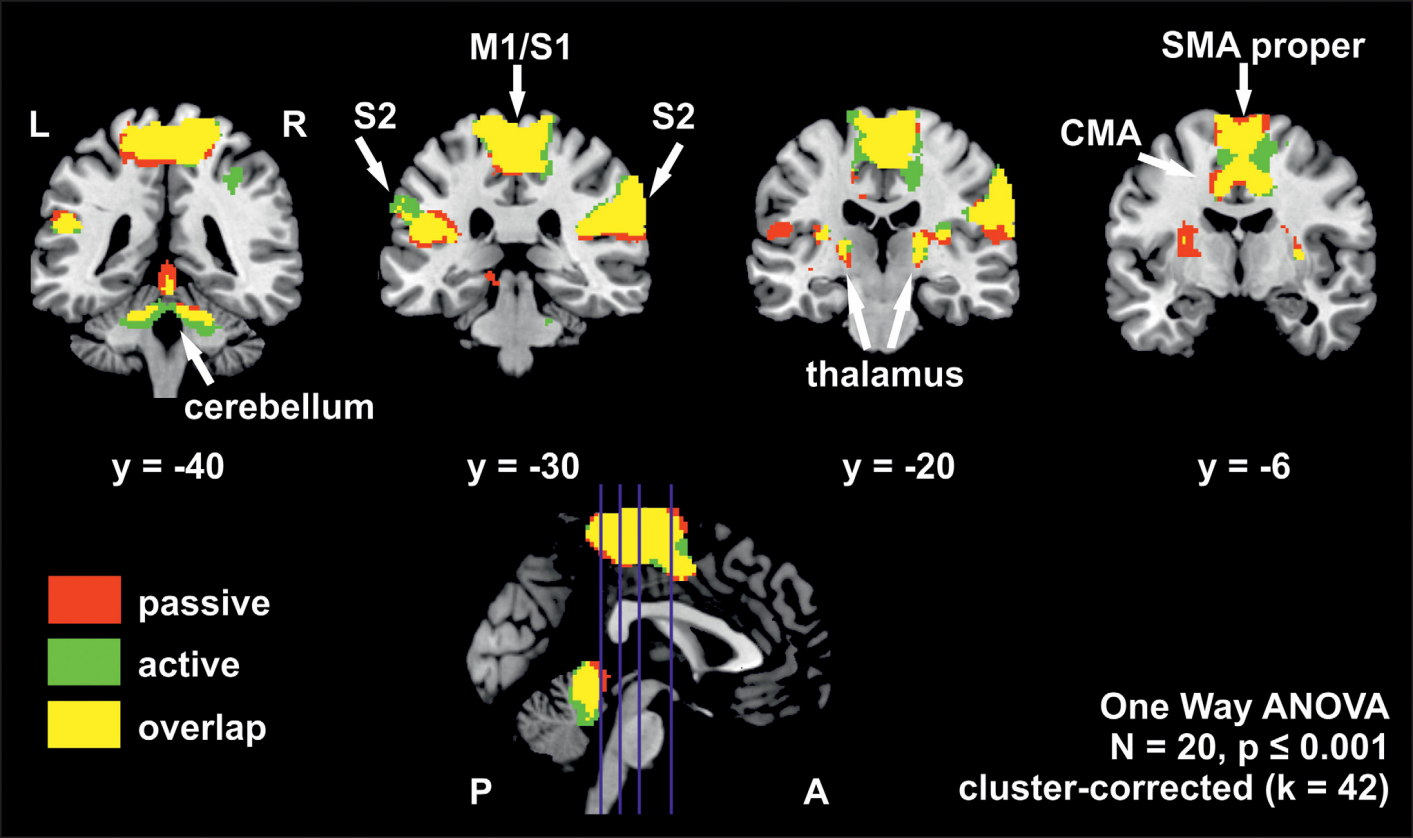
**Overlay of BOLD-signal during active (green) and passive (red) stepping reveals robust activations in an extended sensorimotor network**. Overlapping activations in yellow. The positions of the coronal slices are indicated by the blue lines in the sagittal slice at the bottom. Estimated β-weights and percent signal changes from the ROI-analysis are provided in Table 4. M1/S1, primary sensorimotor cortex; S2, secondary sensory cortex; CMA, cingulate motor area; SMA proper, supplementary motor area proper; L, left hemisphere; R, right hemisphere; P, posterior; A, anterior.

The whole brain group analysis of fMRI BOLD data revealed significant signal changes during active as well as during passive stepping relative to baseline. Both conditions activated an extended cortical and sub-cortical set of regions in the sensorimotor network bilaterally (voxel threshold *p* < 0.001, cluster-corrected, *k* ≥ 42 voxels). This set of regions comprised an extended cluster of activated voxels covering the paracentral lobules, composed of bilateral medial M1/S1 areas (including the areas for knee, ankle and foot in Kapreli et al., 2006), SMA proper, CMA and S2. Additional activations were seen in the anterior cerebellar vermis, cerebellar hemispheres, thalamus, and right putamen (Figure 2, Table 3).

**Table 3:** **Cortical and sub-cortical regions of significant activation during passive, active, passive vs. active and active vs. passive stepping**.

**Anatomy**	**Left hemisphere**					**Right hemisphere**				
	***x***	***y***	***z***	***t***	**Area**	***x***	***y***	***z***	***t***	**Area**
**PASSIVE**										
SMA proper	−2	−14	64	9.23	6	6	−18	64	8.42	6
SPL	−16	−48	64	7.21	7a	14	−50	64	7.75	5l
M1	−10	−42	68	6.53	4a	1	−32	60	9.34	4a
S1	−20	−42	62	6.01	2	16	−36	72	5.59	3
CMA	−4	−2	46	5.76	24	12	−2	42	7.09	24
Supramarginal gyrus	−54	−36	28	5.51	40, OP1	46	−30	24	9.36	OP1
Putamen	−28	−6	12	5.55		30	2	6	6.51	
Insula	−32	−24	14	5.33	13, Ig1	32	−20	12	5.16	Ig2
Thalamus	−18	−24	0	4.05	VPL	18	−20	10	5.27	VPL
Vermis	–	–	–	–		0	−46	−8	7.1	Anterior
Cerebellum	−18	−38	−28	4.52	Anterior	18	−38	−26	5.11	Anterior
Inferior temporal gyrus	–	–	–	–		46	−62	−4	4.82	37
Middle temporal gyrus	−46	−64	0	3.94	37	46	−70	4	3.38	37
Lingual gyrus	–	–	–	–		16	−78	−12	4.54	18
Superior occipital gyrus	–	–	–	–		18	−102	14	4.49	18
Calcarine gyrus	–	–	–	–		10	−92	6	3.63	17
Middle occipital gyrus	−52	−70	−2	3.7	37	30	−84	8	3.37	18
**ACTIVE**										
SMA proper	−2	−14	64	9.74	6	2	−6	52	7.63	6
CMA	−8	−2	40	6.79	24	8	−6	42	6.8	23
M1	−8	−26	62	8.55	4a	2	−32	62	8.93	4a
SPL	−16	−50	64	7.42	5	14	−48	60	5.97	5
Supramarginal gyrus	−58	−34	32	4.68	IPC (PF)	68	−26	34	8.43	2,40, OP 1
Putamen	–	–	–	–		30	0	8	6.26	
Thalamus	−20	−22	8	5.19	VPL	18	−20	8	5.77	VPL
Insula	−46	−2	8	5.28		44	2	6	5.18	13
Insula	−32	−24	14	5.25	Ig1	–	–	–	–	
Vermis	−6	−44	−24	8.33	Anterior	2	−46	−12	7.36	Anterior
Cerebellum	−30	−74	−20	4.44	Posterior	–	–	–	–	
Inferior occipital gyrus	−48	−76	−4	4.13	19	–	–	–	–	
Middle occipital gyrus	−40	−84	2	4.52	19, V5/MT	46	−74	4	5.42	19
Cuneus	–	–	–	–		16	−92	14	4.18	18
Lingual gyrus	–	–	–	–		8	−82	−8	4.17	18
**PASSIVE VS. ACTIVE**										
Olfactory cortex	−18	6	−14	6.54		22	12	−12	6.34	
Superior frontal gyrus	−14	56	28	6.23	9, 10, DLPFC	22	50	18	5.17	10, APFC
Superior medial gyrus	–	–	–	–		8	38	46	5.09	
Middle frontal gyrus	−28	54	6	5.9	10, APFC	–	–	–	–	
Pre-SMA	−14	14	64	4.43	6	–	–	–	–	
PMC	−26	12	60	4.84	6	12	24	62	4	6
Anterior cingulate cortex	–	–	–	–		6	48	12	5.43	
Parahippocampal gyrus	−10	−6	−20	5.43		–	–	–	–	
Putamen	−20	6	6	4.35		22	8	−10	5.71	
Caudate nucleus	−16	12	12	4.18		20	16	8	4.71	
Amygdala	−26	−6	−14	3.66		22	−4	−16	5.44	
Posterior cingulate cortex	–	–	–	–		6	−56	30	5.69	
Middle cingulate cortex	−6	−44	36	5.02	23	4	−44	32	4.96	23
Angular gyrus	−40	−72	38	5.41	19, IPC	52	−70	36	5.35	39, IPC
Middle temporal gyrus	−60	−10	−22	5.34	21	54	−8	−16	4.53	21
Superior temporal gyrus	−46	−18	−6	4.08		60	−10	−10	4.98	22
Parahippocampal gyrus	−16	−34	−8	5.33		10	−30	4	4.74	
Cerebellum	–	–	–	–		18	−84	−30	4.04	Posterior
**ACTIVE VS. PASSIVE**										
Vermis	−8	−42	−26	5.36	Anterior	8	−44	−26	4.1	Anterior
SMA proper	−1	−6	56	4.17	6	6	−4	54	4.3	6
Supramarginal gyrus	–	–	–	–		38	−36	36	4.2	2

All coordinates are in MNI-space, voxel threshold *p* ≤ 0.001, cluster-corrected, *k* = 42 voxels. SMA, supplementary motor area; SPL, superior parietal lobe; M1, primary motor cortex; S1, primary somatosensory cortex; CMA, cingulate motor area; PMC, premotor cortex.

The ROI analysis from the conjunctly activated areas during the active and passive condition revealed significantly higher β-weights and percent signal change for active stepping in bilateral S1/M1, bilateral SMA proper and the cerebellar vermis. A trend for higher activation during active than passive stepping was found for bilateral CMA as well as left and right S2 (for *p*-values, see Table 4).

**Table 4:** **β-weights and percent signal change of the analyzed ROIs**.

**Area**	**β-weights**								**% signal change**							
	**Passive**			**Active**				**Effect size**	**Passive**			**Active**				**Effect size**
	**Mean (*SD*)**	**Min**	**Max**	**Mean (*SD*)**	**Min**	**Max**	***p*-value^1^**	**Cohen's d**	**Mean (*SD*)**	**Min**	**Max**	**Mean (*SD*)**	**Min**	**Max**	***p*-value^1^**	**Cohen's d**
bil. M1/S1	0.093 (0.088)	−0.124	0.237	0.142 (0.080)	−0.027	0.327	0.014^**^	0.58	0.330 (0.303)	−0.42	0.83	0.497 (0.292)	−0.12	1.21	0.014^**^	0.56
bil. SMA proper	0.216 (0.160)	−0.184	0.469	0.318 (0.158)	0.080	0.619	0.007^**^	0.64	0.810 (0.605)	−0.69	1.67	1.216 (0.614)	0.28	2.38	0.006^**^	0.66
bil. CMA	0.050 (0.042)	−0.028	0.135	0.070 (0.045)	−0.014	0.149	0.086^*^	0.46	0.197 (0.163)	−0.11	0.49	0.275 (0.178)	−0.05	0.59	0.079^*^	0.45
l.S2	0.079 (0.066)	−0.050	0.198	0.095 (0.085)	−0.106	0.254	0.086^*^	0.21	0.281 (0.233)	−0.17	0.73	0.328 (0.304)	−0.46	0.85	0.093^*^	0.17
r.S2	0.084 (0.054)	−0.031	0.176	0.110 (0.068)	0.016	0.298	0.073^*^	0.42	0.314 (0.203)	−0.11	0.68	0.411 (0.256)	0.06	1.12	0.067^*^	0.42
Cerebellar vermis	0.132 (0.066)	0.000	0.283	0.203 (0.170)	−0.316	0.487	0.014^**^	0.55	0.379 (0.194)	0.00	0.81	0.563 (0.488)	−1.03	1.35	0.014^**^	0.50
r. putamen	0.039 (0.024)	0.005	0.078	0.046 (0.041)	−0.019	0.181	0.881	0.21	0.153 (0.092)	0.02	0.30	0.183 (0.162)	−0.07	0.73	0.940	0.23
l.IOG	0.090 (0.162)	−0.330	0.441	0.133 (0.121)	−0.017	0.474	0.218	0.3	0.340 (0.605)	−1.19	1.67	0.485 (0.418)	−0.06	1.52	0.263	0.28
r.MOG	0.100 (0.166)	−0.263	0.519	0.136 (0.133)	−0.105	0.574	0.117	0.24	0.362 (0.616)	−0.99	1.94	0.472 (.442)	−0.39	1.87	0.167	0.21
r.ITG	0.100 (0.134)	−0.189	0.421	0.153 (0.136)	−0.016	0.624	0.126	0.39	0.365 (0.494)	−0.69	1.58	0.544 (0.472)	−0.06	2.14	0.126	0.37
r.LG	0.166 (0.227)	−0.269	0.712	0.210 (0.200)	−0.203	0.592	0.204	0.21	0.600 (0.810)	−0.96	2.41	0.724 (0.689)	−0.72	2.02	0.263	0.17

*Values are group means (*SD*). bil., bilateral; l, left hemisphere; r, right hemisphere; M1/S1, primary sensorimotor cortex; SMA proper, supplementary motor area proper; CMA, cingulate motor area; S2, secondary somatosensory cortex; IOG, inferior occipital gyrus; MOG, medial occipital gyrus; ITG, inferior temporal gyrus; LG, lingual gyrus*.

1 *Wilcoxon signed ranks test* (α = 0.05),

** *p* ≤ 0.05,

* *p* ≤ 0.1.

The correlation analysis between the ROI β-weights and the performance metrics did not yield any statistically significant correlations between the degree of brain activation and participant performance (*p*-values > 0.05).

#### Active vs. passive stepping

The contrast of active vs. passive stepping yielded significant activation in the SMA proper, the anterior vermis of the cerebellum and the right supramarginal gyrus.

#### Passive vs. active stepping

The contrast passive vs. active stepping revealed clusters of significant BOLD signal changes in the bilateral medial prefrontal gyrus, anterior and posterior cingulate gyrus, angular gyrus, inferior parietal cortex, parahippocampal gyrus, and anterior vermis of the cerebellum.

**Figure 3:**
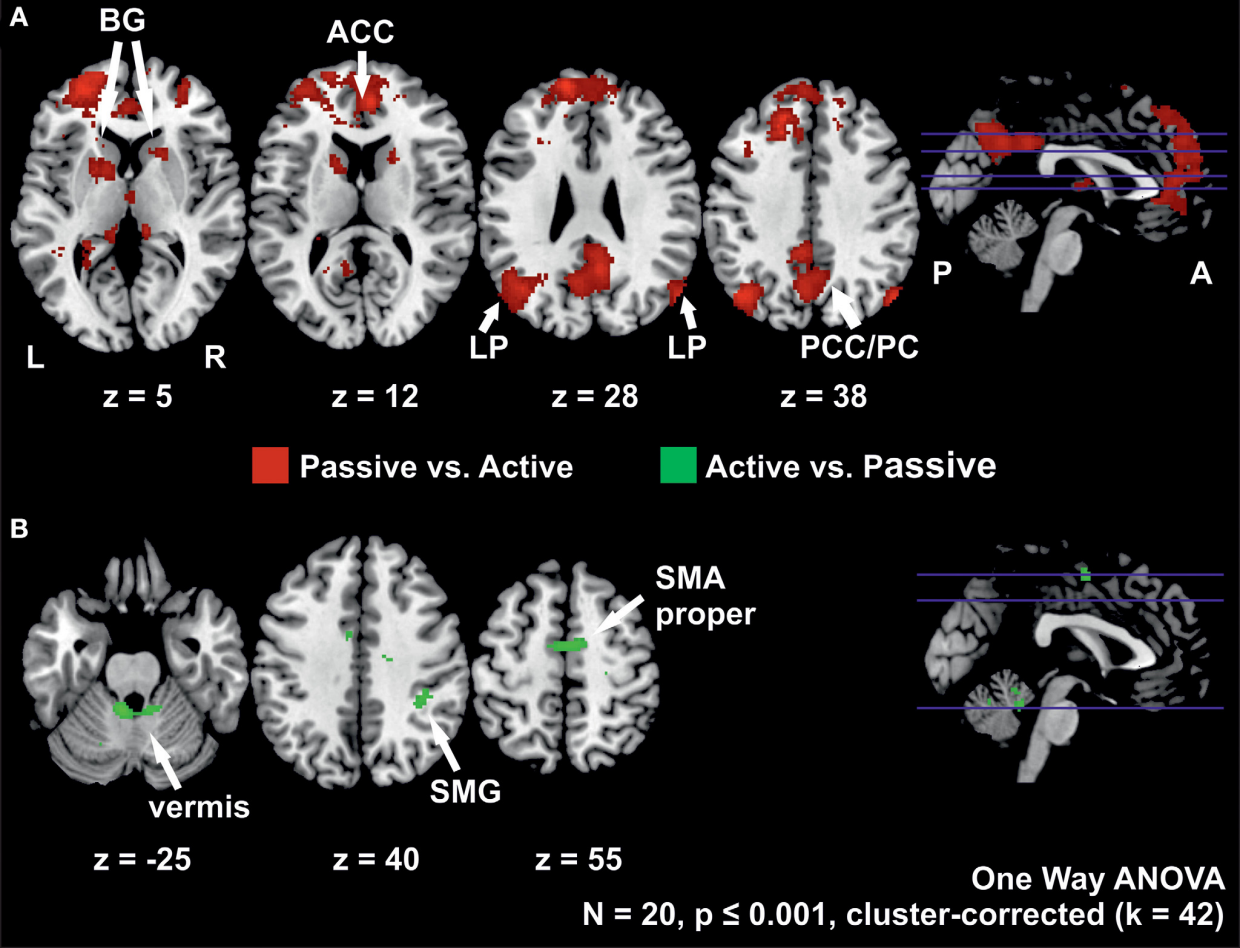
**(A)** Clusters with higher activation during passive than during active stepping and **(B)** activation higher during active than during passive stepping. The positions of the axial slices are indicated by the blue lines in the sagittal slice on the right; BG, basal ganglia; LP, lateral parietal cortex; ACC, anterior cingulate cortex; PCC/PC, posterior cingulate cortex/precuneus; SMG, supramarginal gyrus; SMA proper, supplementary motor area proper; L, left hemisphere; R, right hemisphere; P, posterior; A, anterior.

Further areas activated in this contrast include bilateral pre-motor cortex and left pre-SMA as well as bilateral basal ganglia (putamen and caudate nucleus) (Figure 3, Table 3).

## Discussion

The aims of the present study were (a) to demonstrate the feasibility of a novel imaging paradigm combining the MR-compatible stepper MARCOS with a sparse temporal sampling fMRI protocol, and (b) to delineate the supraspinal contribution specific to active and passive, bilateral, periodic, multi-joint, lower limb motor control in healthy participants. Our data revealed significant, task-correlated neural activation in a congruent set of regions in the sensorimotor network during active and passive stepping. The ROI-analysis demonstrated higher activation (i.e., β-weights and percent signal change) of these regions for active than for passive stepping. No significant correlations between the degree of brain activation and participant performance were found.

### Congruent yet differential activation of sensorimotor areas during active and passive stepping

The present study yielded significant activation in cortical and sub-cortical bilateral sensorimotor areas including medial M1/S1, SMA proper, CMA, S2, cerebellar vermis and putamen associated with rhythmic, reciprocal active and passive stepping akin to human locomotion.

Substantial overlap of neural activations in these areas between active and passive movements of the lower limbs has been reported in the past, both for unilateral ankle movements (Dobkin et al., 2004; Ciccarelli et al., 2005; Francis et al., 2009) as well as for bilateral multi-joint tasks of the lower limbs (Christensen et al., 2000; Mehta et al., 2012). Since the strength of activity in M1/S1 and SMA was not significantly different between active and passive pedaling in the study of Mehta et al. (2012), this activity was suggested to represent the monitoring of ascending proprioceptive afferents rather than the production of descending efferents to the lower limb muscles required for active movements (Mehta et al., 2012). However, this assumption is challenged by our finding of a significantly higher degree of brain activation (i.e., β-weights and percent signal change) in several sensorimotor areas, i.e., bilateral medial M1/S1, SMA proper and cerebellar vermis and a trend for higher activation in bilateral CMA and S2, during active movement execution. Higher peak activations in M1/S1, SMA, the cerebellum and the putamen during active unilateral ankle movement (Dobkin et al., 2004; Ciccarelli et al., 2005) and increased cerebral blood flow in M1 during active bicycling (Williamson et al., 1997) have been reported in previous studies as well.

The cerebellar vermis receives projections from the motor cortex (Coffman et al., 2011) and is known to be intimately involved in adaptation and interlimb coordination during locomotion by modulating timing, rate and force of muscle activity (Morton and Bastian, 2006, 2007). Gait ataxia is a prominent and notorious symptom of cerebellar dysfunction. The SMA has repeatedly been involved in bimanual (Swinnen, 2002) and interlimb (i.e., hand-foot) coordination (Heuninckx et al., 2008) and becomes increasingly activated when more difficult spatial relations between simultaneous limb movements have to be monitored (Debaere et al., 2001). This is supported by the finding that the temporal control of bimanual coordination is deteriorated when SMA is inhibited by repetitive transcranial magnetic stimulation (Obhi et al., 2002; Serrien et al., 2002). We suggest that the relatively higher activation of these sensorimotor areas and their more pronounced activity of the cerebellar vermis and the SMA contribute to the generation of sequentially descending motor commands and to the increased demands in temporal and spatial coordination of bilateral active as compared to passive stepping movements. This is further supported by the fact that the studies by Mehta et al. (2012) and Christensen et al. (2000), which found largely the same activity across active and passive movements, used a constant phase-shift between the legs during cycling movements. The opposed and rigid arrangement of the cranks of their devices set the phase shift and hence the timing of muscle activation between the two legs. In contrast, stepping inside the robot MARCOS requires a higher coordinative effort to maintain a phase difference of 180° between the moving limbs during the active but not the passive condition, as the phase shift is not dictated by the robot during active. This might further have contributed to the observed between condition differences in the somatosensory system in present study.

Differences in the neural representation of active and passive lower limb motor control are further corroborated by the contrasts of active vs. passive and passive vs. active stepping. Active vs. passive stepping revealed significant bilateral activation in the anterior cerebellar vermis, and SMA proper, and the right supramarginal gyrus. These areas are a subset of the neural activity generally reported in studies of active vs. passive unilateral ankle dorsiflexion (Sahyoun et al., 2004; Ciccarelli et al., 2005; Francis et al., 2009). These reports demonstrated a more distributed set of regions for the contrast active vs. passive, including M1/S1, CMA, PMC and subcortical structures (Table 5). The confined activations for active vs. passive in the present study could be the result of differences in task complexity (i.e., bilateral multi-joint vs. unilateral single-joint movements).

**Table 5:** **Overview of fMRI studies investigating active and passive unilateral ankle-dorsiflexion and their main findings for active vs. passive movements at the whole brain level**.

**Study**	**Main findings**
Present study	High degree of overlap of activations between active and passive movement in M1/S1, SMA proper, CMA and S2. Additionally in the anterior cerebellar vermis, both cerebellar hemispheres, thalamus, and right putamen.
	Activations in M1/S1, SMAproper and Vermis significantly stronger during active than during passive, trend for stronger activation during active in CMA and S2.
	Active vs. passive: Active movement generated significantly stronger activation in SMA proper, the anterior vermis of the cerebellum and the right supramarginal gyrus.
Sahyoun et al., 2004	High degree of overlap of activations between active and passive movements in M1/S1, SMA, and S2.
	Activations in the M1/S1, SMA, and PMC were consistently stronger during active than passive movements.
	Active vs. passive: stronger activation in M1/S1, SMA, PMC, CMA, cerebellum, putamen, thalamus, insula, and inferior frontal gyrus.
Ciccarelli et al., 2005	Overlap of significant activations between active and passive movements in contralateral M1/S1, SMA, bilateral rolandic operculum and insula, ipsilateral superior temporal gyrus, ipsilateral cerebellum, and contralateral posterior putamen.
	Active vs. passive: stronger peak activations in M1/S1, SMA, cerebellum, putamen, superior temporal gyrus, inferior parietal lobe.
Francis et al., 2009	Active and passive movements activated M1/S1, putamen, SMA and CMA, bilateral S2, insula, ipsilateral PMC.
	Active vs. passive: stronger activation in SMA, contralateral M1/S1, SII and CMA, bilateral PMC and cerebellum.

M1/S1 = primary sensorimotor cortex, SMA = supplementary motor area, S2 = secondary sensory cortex, PMC = premotor cortex, CMA = cingulate motor area.

The contrast of passive vs. active on the other hand, revealed increased bilateral activation in a distributed set of regions in the medial and lateral frontal lobe and inferior parietal lobe, as well as subgenual ACC. These areas have also been reported by Sahyoun et al. (2004) in their comparison of passive vs. active unilateral ankle movements, albeit to a smaller spatial extent, probably related to the lower complexity of their task (i.e., unilateral movement of a lower number of joints). These findings, however, are at variance with the PET study of Weiller et al. (1996) who reported activation of bilateral S2 characteristic of unilateral passive elbow movement when compared to unilateral active elbow movement. Other studies on active and passive motor control do not report on the direct contrast of passively vs. actively generated movement.

In the present study, subjects had to attentively monitor and maintain limb inactivity while perceiving the passive movements. This required the voluntary cortical inhibition of a motor response to a degree where no peripheral muscular activation was produced. Furthermore, eventual spinal reflexes elicited by passive movement of the limbs had to be inhibited by adequate descending supraspinal commands.

Our finding of activation in the subgenual ACC, accompanied by activation in the anterior medial prefrontal cortex, has also been described during motor response inhibition in a Go/No-Go task by (Liddle et al., 2001), in which the subgenual ACC was assigned a role in monitoring of the “internal state,” while the (anterior medial) prefrontal cortex was seen as a protagonist in response inhibition. A functional interplay between the ACC with the anterior medial prefrontal cortex is further supported by findings of functional connectivity between these two areas (Margulies et al., 2007). Our spatial locations of activations for the contrast passive vs. active do overlap with the spatial locations commonly activated by other motor response inhibition experiments (Rubia et al., 2001). The study by Rubia et al. (2001) reported additional activation of preSMA and medial and lateral parietal lobe during motor response inhibition. The authors highlighted the role of these areas in higher-order motor function monitoring such as motor attention or response selection while inhibiting a motor response. In conclusion, we suggest that the activations accentuated in the contrast of passive vs. active stepping rather reflect the cognitive processes required for the maintenance of limb passivity (i.e., the inhibition of an active motor response) than the mere integration of somatosensory afferent input.

With regards the differences in the level of activation between active and passive stepping we believe that the results of the ROI analysis are not confounded by circularity (i.e., double dipping). Eight of the ROIs were defined using a conjunction of the contrasts active vs. baseline and passive vs. baseline (Nichols et al., 2005). This approach was suggested also in the supplementary discussion of Kriegeskorte et al. (2009). Furthermore, the definition of three ROIs (i.e., S1/M1, SMA-proper, and CMA), based on anatomical coordinates reported for knee movements (Kapreli et al., 2006), i.e., a “selection bias” is precluded for these ROIs.

Yet, for the observed activation of the olfactory cortex (contrast: passive vs. active) we cannot offer a concise physiological explanation. We suspect it to be produced by residual head movement (i.e., not dealt with by the sparse-sampling design), and thus, it occurred as the result of the spatial proximity of the olfactory cortex to the base of the skull, an area which is known to be susceptible to movement artifacts.

Since EMG was not measured during task execution in the present study, it cannot be completely ruled out that some of the neural activity observed during passive movement is caused in part by involuntary muscle activation. However, a recent study by our group demonstrated that healthy participants are very well able to remain passive while MARCOS is moving their legs along the predefined trajectory (Marchal-Crespo et al., 2014). It is safe to assume that this was also the case in the present study, since the participant population is comparable to the one in Marchal-Crespo et al. (2014).

#### Topography of peak activations during active movements

Although the motor task in the present study involved simultaneous and bilateral movements about of the hip, knee and ankle joints, we compared the spatial locations of peak activations in several areas during active movements to those reported in a previous somatotopy study involving the same body parts. Generally, our peak activations were located in the vicinity of those reported by Kapreli et al. (2006), a study which investigated BOLD signal changes in response to unilateral and isolated knee, ankle, and toe movements. In the M1/S1 region, our peak activation was located more medially to the one reported for the knee and lateral to the ankle and the toe. In both, the SMAproper and the CMA our peak activations were located anterior and inferior to those reported in Kapreli et al. (2006). PMC activations were in general located medial and anterior to those reported for knee, ankle, and foot. The spatial location of activation in the vermis was located in the midline and slightly anterior to isolated knee movements. Similarly, peak activations in the anterior cerebellar hemispheres were located medial to those reported during knee movements.

However, the comparability of spatial locations of peak activations during bilateral, reciprocal movements of the whole legs with isolated, unilateral single-joint movements is limited by differences in the level of task complexity (i.e., bilateral multi-joint vs. unilateral single-joint movements). Additionally, it must be considered that the knee and the foot of each leg are mechanically coupled when participants are secured to the robot MARCOS. The target movement (flexion and extension of the whole leg) could theoretically be achieved through different strategies. The same movement can be realized with either emphasizing hip flexion/extension, and the activation of related muscles or alternatively by accentuating knee flexion/extension. This may have hence influenced the individual and group peak activations.

### Standardization and repeatability of the motor task and their influence on brain activation

The two-way repeated measures ANOVA of task performance (factors “trial” and “condition”) as recorded by the position and force sensors of the robot did not reveal a significant main effect of “trial” for any of the performance metrics in both movement conditions. It is therefore a valid claim that the motor paradigm presented in the current study enables repeatability within active and passive movement conditions. For the passive condition this was expected, since the movements of the lower limbs were brought about by the robotic device. As for the active condition the applied measures to limit knee movement amplitude and movement frequency have proofed to be feasible means to reach high between-trial repeatability.

However, the Two-Way repeated measures ANOVA of task performance also revealed a trend for a significant main effect of condition in movement kinematics (i.e., knee amplitude) and a significant main effect of condition in movement dynamics (i.e., robot-participant mean peak interaction forces at the knee *KF* and foot *FF*) between active and passive stepping movements. From studies in the upper limbs it is known that the degree of brain activation may depend on the exerted force and movement amplitude (Ehrsson et al., 2001; Keisker et al., 2009; Sulzer et al., 2013). However, given the fact that current literature is scarce regarding the influence of lower limb movement dynamics on supraspinal activation patterns, the relevance of the observed differences on brain activation patterns must be interpreted with caution.

The stepping movement amplitude at the knee during active stepping was marginally bigger than during passive. Movement amplitude dependent activation of S2 and the putamen have been described for amplitude differences as small as 0.01 m in passive thumb-index finger opposition movements (Sulzer et al., 2013). The same difference [0.012 m (0.153 m during active vs. 0.141 m during passive)] has been observed in the current study which corresponds to approximately 1.2° difference in hip angle (approximately 17.1° in active vs. 15.9° in passive). This difference is below the accuracy of lower limb motor control during walking as seen by the reported step-to-step variability of human gait (~1.5° at the ankle, ~1.8° at the hip and ~1.9° at the knee) (Winter, 1984). It can therefore be assumed that this trend for a main effect of condition between active and passive movements did not affect the neural activation patterns.

The absolute movement amplitude at the knee was defined before the start of the study, in order to standardize the movement range across all participants. However, to match supraspinal proprioceptive afferent input from length sensitive receptors (i.e., muscle spindles) in the lower limbs, it would have been more intuitive to normalize knee movement amplitudes to individual leg segment lengths across participants. It can be assumed that joint angles rather than end effector positions in space are the variables controlled by and influencing the activity of the human motor system (Miall and Wolpert, 1996). While relative knee movement amplitudes could have been calculated for each participant from individual leg segment lengths using trigonometry with little effort, this would also have required very precise alignment with the robot which was not practicable with all participants. As misalignments would have led to a similar variability in knee movement amplitude as observed in the current data, the same predefined absolute amplitude was chosen for all participants.

Furthermore, significant main effects of condition were found in the mean peak interaction forces *FF* and *KF*, respectively. On average, *KF*_active_ was 73 N higher than *KF*_passive_. Differences in the interaction forces were expected and can be explained by the fact that participants rested the weight of their lower limbs on the robot during passive movements (*KF*_passive_ = −18 N) as the device drove their knees up and down when producing the stepping pattern, while producing forces in the opposite direction when performing active stepping movements. Accordingly, higher mean *FF* were measured during active than during passive stepping (Δ = 16 N). There are currently only two studies investigating the influence of external axial loads to the lower limbs on supraspinal activation (Christensen et al., 2000; Miyai et al., 2006). Both investigated force increments of about 60–80 N (i.e., approximately 10% of body weight) and neither reported area-specific effects on brain activation. This is most likely because the investigated force increments were in the realm of step-to-step variation of vertical ground reaction force during ground-level gait of 7% of individual body weight (Winter, 1984). At the same time support for differential, force-dependent neural activation of brain areas can be found in studies reporting a positive correlation between the activation strength of the sensorimotor network and the gripping forces produced by the hand (Keisker et al., 2009). For the hand, differences in neural activation were described for force increments as little as 14 N (Ehrsson et al., 2001). However, it must be taken into account that the motor tasks performed by the upper and lower extremities are very specific (i.e., grasping vs. stepping) and require different accuracy in position and force control. It is therefore likely, that the sensory threshold eliciting differential supraspinal activation within the same type of movement is intrinsic to the extremity in use. With regards to the differences in interaction force between the robot and the participants in the current study, we therefore assume that they did not affect the supraspinal activation patterns.

Furthermore, no significant correlations between the level of brain activation and participant behavior could be demonstrated. This supports our assumption that neither the trend for a statistically significant difference in movement amplitude KA nor the significant differences in interaction forces *KF* or *FF* lead to a detectable influence in the supraspinal activation patterns.

### Head motion despite clustered sparse sampling imaging protocol

Head-motion concurrent to image acquisition, i.e., the movement of brain tissue into a voxel of different magnetic field intensity, is a well-known issue of *in-vivo* neuro-imaging and is particularly pronounced in studies related to brain activation during lower limb motor control. It has been shown, that movements typically propagate along the caudo-cranial body axis while the lower-limbs are being moved (Seto et al., 2001). These displacements can lead to changes in the magnetic susceptibility of the tissue (Seto et al., 2001), introducing considerable confounding effects on signal intensity, i.e., spin-history effects (Friston et al., 1996), and lead to false positive activations not recognizable as such on activation maps (Field et al., 2000). In addition, the homogeneity of the static magnetic field may become distorted by moving body parts, thus introducing further perturbations during image acquisition (Mehta et al., 2009; Lemmin et al., 2010). In this study, a clustered sparse temporal sampling acquisition scheme was applied, exploiting the temporally sluggish nature of the BOLD signal. The sequential arrangement of task execution and image acquisition temporally separated leg motion from data collection resulting in only non-task related trial-to-trial head motion in the imaging data. Lower statistical power is traded in for less noisy and blurred signals. To our knowledge there are currently only two studies that exploited sparse temporal sampling to investigate sensorimotor control (Dresel et al., 2005; Toyomura et al., 2012). In the pedaling studies of Mehta et al. (2009) and Mehta et al. (2012) fMRI data were acquired continuously over the course of the experimental runs, but analyzed only between movement trials to minimize movement related artifacts. Methodologically, this “delayed” analysis approach conforms to the analysis performed on the sparse sampling data of our study and yielded compatible results.

The gain in reduced sensitivity to task-correlated head motion comes at the expense of certain disadvantages as compared to continuous sampling. Firstly, measuring the BOLD signal only after the cessation of the motor task hinders the analysis of brain activation of specific phases of the motor task (e.g., movement preparation or movement initiation). Secondly, the T1-decay along the consecutive volumes may alter the contrast of EPI images potentially affecting the accuracy of the fMRI data preprocessing steps that depend on tissue contrast (Perrachione and Ghosh, 2013). Nevertheless, given the large amount of head motion and the magnetic field inhomogeneity associated with stepping movements, the benefits of the chosen sparse sampling approach justify its application for BOLD imaging during the specific experimental conditions used in our study. A reliable and valid analysis of imaging data would otherwise be very limited.

### Possible implications for robot aided gait rehabilitation of neurologic patients

Although our study investigated healthy participants, our findings may have direct implications for the management of gait impaired patients, for the development and application of robotic rehabilitative devices in the clinical setting, and for the monitoring of brain reorganization using standardized and repeatable experimental procedures over time.

The activation of the sensorimotor network during passive movement of the participants' legs indicates that the ascending proprioceptive feedback from passive movement alone suffices to stimulate the same supraspinal cortical and sub-cortical sensorimotor areas as active movements, although to a lesser degree. The peripheral sensory cues produced by passive movement might be a suitable rehabilitation strategy for severely motor-impaired patients with very limited, or absent voluntary activation of their lower limb muscles, firing up the processes of brain reorganization and maintaining brain function in de-efferented patients. Furthermore, repeated passive movement allows the investigation of motor control related brain areas in these patients who cannot move their limbs. A possible issue with passive movement in gait rehabilitation however is, that patients with some residual voluntary activity might soon rely completely on the robot and lose their self-motivation for, and attention to the task if not challenged appropriately (Wolbrecht et al., 2008). The increased temporal and spatial complexity of motor coordination and the required voluntary muscular activity in the lower limbs during active task execution increased the level of brain activation in our study (i.e., significantly higher β-weights and percent signal change during active than during passive movements). In rehabilitation, less disabled patients should be challenged with sophisticated rehabilitation strategies that only assist their movements as needed and adapt their support to the abilities of the individual patient (Harwin et al., 2006; Duschau-Wicke et al., 2010; Schuck et al., 2012; Badesa et al., 2013), such that optimal involvement and activation of the brain can be achieved. The robot MARCOS provides the possibility to implement such supportive control algorithms and the opportunity to directly investigate their effects on brain activity in fMRI experiments (Hollnagel et al., 2013).

## Conclusion

We conclude that the combination of a clustered sparse temporal fMRI protocol with the MR-compatible stepper MARCOS is a feasible instrument for the standardized and repeatable investigation and detection of the neural correlates of active and passive, bilateral, lower limb stepping movements akin to human locomotion. Significantly higher activation of the sensorimotor network during active than during passive bilateral lower limb motor control may represent more than the mere monitoring of afferent proprioceptive input.

Although our study investigated healthy participants, the presented paradigm should enable future work with neurologic gait-impaired patients in cross-sectional and longitudinal studies. Clinical rehabilitation studies combining gait training with standardized and repeatable neuro-imaging experiments of gait-like stepping may yield more accurate knowledge about effects of specific training strategies on supraspinal activation patterns and thereby support the development and application of novel approaches to motor rehabilitation.

### Conflict of interest statement

The authors declare that the research was conducted in the absence of any commercial or financial relationships that could be construed as a potential conflict of interest.
